# What is the *point* of a base system? Denotation, computation and the non-integers

**DOI:** 10.1098/rstb.2024.0219

**Published:** 2025-10-20

**Authors:** Oisín Parkinson-Coombs, Rafael Núñez

**Affiliations:** ^1^ D-GESS, ETH Zürich, Zürich, Switzerland

**Keywords:** base, decimal system, decimal fractions, mathematical notation, history of mathematics, decimal point

## Abstract

As researchers and as laypeople, we inhabit worlds replete with numerals, numerals structured by a base system. We rely on exponentiation and powers of the base to describe these systems. This research habit, along with our familiar experience of numerals as expressing precision, produces a conceptual blind spot regarding their development, structure and purpose. In examining the protracted historical developments of the decimal base system for non-integers—which (i) emerged hundreds of years after the integer positional system, (ii) relied on tools developed in different domains separate from concerns of precise denotation, and (iii) were adopted owing to contingent computational benefits—we see that this blind spot obscures our understanding of base, exponentiation and the non-integers. The usage of exponentiation in the structure of ‘base’ and positional systems emerged as a result of this extension rather than through the explication of this structure. Although lingua francas are important in research, we must be sensitive to their history, especially when their structure and purpose in contemporary contexts may differ from their historical and developmental contexts.

This article is part of the theme issue ‘A solid base for scaling up: the structure of numeration systems’.

## 1. Mathematical nomenclature in numerical research

The concept of ‘base’, as applied to the study of numeral systems, contains multitudes. Borrowing the term from mathematics, in linguistics, ‘base’ has been coarsely used to classify numeral systems with apparently distinct compositional rules [1]. In Pelland’s [2] philosophical discussion of ‘base’, he highlights its intimate ties with counting and a recursive counting unit. In historical work, the term ‘base’ has been used largely in the context of place value notational systems (see [3]). While intimately connected with these concepts, in modern mathematics, the concept of ‘base’ relies on a network of technical, rich concepts, not limited to compositionality, counting or place value. Take the familiar Indo-Arabic notational numeral system in which any integer can be expressed as


$$\begin{array}{rl} a_{m}\cdots a_{0} & :=\sum_{k=0}^{m}a_{k}\cdot 10^{k},\\ \text{ with }a_{k}\in \{0 & ,\ldots ,9\}\text{ and }m\in N. \end{array}$$

This explicitly relies on the operations of addition, multiplication and exponentiation. In fact, *Encyclopaedia Britannica* explicitly defines ‘base’ in these terms as ‘an arbitrarily chosen whole number greater than 1 in terms of which *any number can be expressed as a sum of that base raised to various powers*’ [4] (emphasis added). Although appearing to be a mere distillation of the elementary concepts structuring the familiar system, such notation and definitions implicate additional features and mathematical constructions that ground their meaning and use. For instance, in modern mathematics, exponentiation is not just repeated multiplications; it is well-defined for all real numbers including zero: $2^{0}=1$; negative numbers: $2^{-1}=0.5$; fractions: $2^{\frac{1}{2}}=\sqrt{2}$ and even irrational numbers: $2^{\pi }=8.824\ldots$

Taking contemporary mathematics to define the relevant explanatory goals of a field is not always appropriate; in the case of ‘base’ in numeral systems, it might appear far removed from what the locus of study ought to be. However, the rich concepts and constructions of contemporary mathematics often get invoked in research on numeral systems, numerical cognition and ‘base’, particularly exponentiation. In numerical cognition, Dehaene invokes the exponential^1^ description of the Indo-Arabic notational numeral system, stating that ‘successive places in the number represent successive *powers of the base*, from units ($10^{0}=1$) …’ [6, p. 98] (emphasis added). In comparative anthropology, Bender *et al*. [7, p. 555] use exponential notation to describe the compositionality of lexical numeral systems:


(1.1) $$N_{gen}=[nP_{10^{x}}]+\cdots +[nP_{10^{2}}]+[nP_{10^{1}}]+[n],$$

and notational numeral systems are described as *multiplicative* if a numeral phrase in that system’s ‘two components *per power*, unit-sign(s) and a *power-sign*, multiplied together, give that *power’s total value*’ [8, p. 13]. It is not necessarily true that exponentiation (or power) is present in the systems being described, any more than in the regular hierarchical system of pecks, bushels and bolls (an obsolete imperial system of dry volumes). Moreover, the development of powers expressed via numerals rather than a hierarchy of disparate glyphs and rhetorical terms (if expressed at all) only emerges in the sixteenth and seventeenth centuries, and originated in the context of repeated multiplications of unknowns rather than of concrete numerals. Although efficient lingua francas are essential for productive research, using mathematical notation and concepts uncritically can distort our understanding of both the concept’s presence or absence in a particular domain and the concept’s historical development. In the case of ‘base’ specifically, this can lead us to tacitly hold that exponentiation is immanent in ‘base’ systems and their use.

## 2. A conceptual blind spot?

The possible negative effects accompanying this mathematical lingua franca can be mitigated by careful historical and typological work. In fact, many of the authors we cite above and throughout this text do just this. By sensitively attending to the historical record, the clash between the casual inferences owing to our mathematical nomenclature and the actual evidence can be noticed and investigated–without having to jettison the communicatively efficient language tout court. However, the use of the mathematical lingua franca can prevent precisely that attention; the notation can obscure subtle differences and leave us with a sense of conceptual continuity where there is discord and diversity [9]. In the case of ‘base’ specifically, by tacitly accepting exponentiation as latently structuring these numeral systems, we can be led to hold that there is a simple continuity between the numeral systems as applied to integers and non-integers. In the *Encyclopaedia Britannica* definition of ‘base’ mentioned above, this continuity is evidenced in the choice of a prototypical example of ‘base’ as a number with a non-integer part expressed using exponentials: ‘The decimal number system that is commonly used expresses all numbers in base 10. For example, $354.76=(3\times 10^{2})+(5\times 10^{1})+(4\times 10^{0})+(7\times 10^{-1})+\left(6\times 10^{-2}\right)$’ [4]. This implicit continuity can also be seen in Ifrah’s classic book, *The universal history of numbers: from prehistory to the invention of the computer* [10], while discussing the formal characterization of positional numeral systems with a base, we read

This positional notation may be *extended easily to fractions with a base power for denominator*, and thus to a simple and coherent notation for all the other numbers, rational and irrational, by dint of a point [10, p. 344] (emphasis added).

Although not making an immediate historical claim in this passage, the supposed ease of this extension, along with the vernacular usage of ‘decimal’ for both integer and non-integer numerals, leaves us with the sense of a continuity; a continuity that only makes sense in the light of describing both the integer and non-integer systems in terms of contemporary exponential (power) concepts and notations.

Relatedly, we live in worlds replete with numerals, with numeral forms shared between the integers and non-integers. Reflect for a moment on your own experience of non-integers. If you are Anglophone, you probably call these ‘decimals’,^2^ the same word you use to describe the positional and lexical numeral systems generally. You probably think of the non-integers, these ‘decimals’, as being used for precision: representing the value of bitcoin, the temperature of the water or the price of your lunch. Style guides frequently caution authors on ‘resisting the *precision of more than one decimal place*’ [11, p.60] (emphasis added) and instruct that authors are ‘discouraged from *implying too much certainty* in their estimated results by offering coefficients that extend to three and four decimal places’ [12] (emphasis added). In quantitative science, we blend the accuracy of measurement with the precision of the notation; *Science* magazine describes advances in atomic clocks as an attempt ‘[to] squeeze *additional decimal points of accuracy* out of the world’s top timekeepers’ [13, p. 1310] (emphasis added).

**Figure 1.**
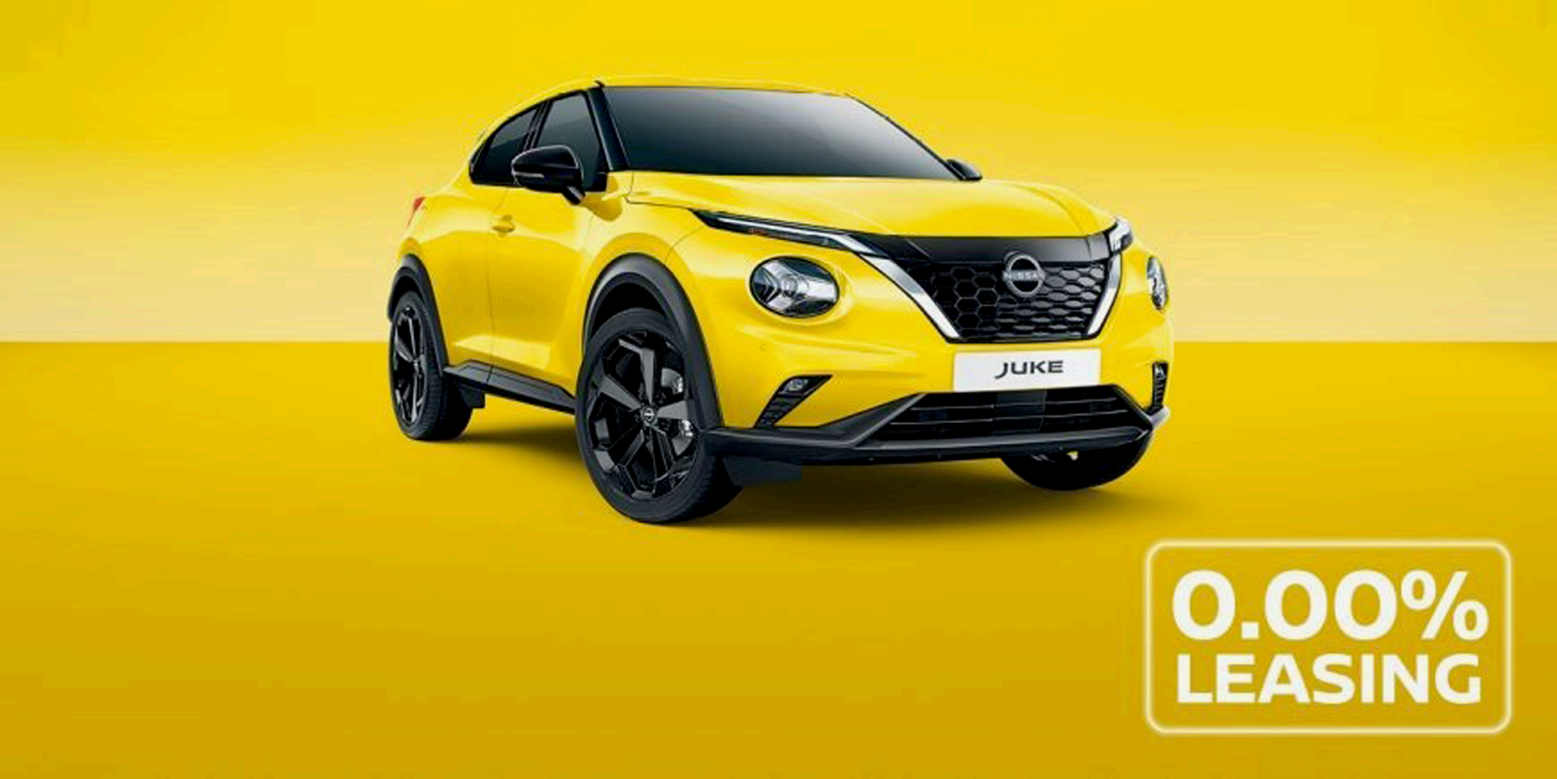
A recent advertisement for the leasing of a car. The advertisement uses the decimal point and two decimal spaces to stress the 0% deal on offer.

In everyday advertising too, the decimal point and trailing digits are used to express precision; in figure 1, the 0% APR deal is emphasized by the 0.00, as though this is somehow more precisely a zero-interest loan than 0% alone. Interacting with numerals for non-integer quantities in these ways elevates denotational precision as their primary function.

As a result, our familiar experience of using decimals for precision, coupled with the implicit continuity we perceive between the decimals and the integers—owing to the shared use of contemporary exponential notation—leaves us with a *conceptual blind spot:*

a sense that *a*) non-integer denotation was merely a straightforward elaboration of positional notation through the extension of its exponential structure, and *b*) was for the purpose of enhancing denotational precision.

For instance, in the popular science magazine *Discover*, we read that

the invention of zero also created a new, *more accurate way to describe fractions*… placing infinitely many digits to the right of the decimal point corresponds to *infinite precision* [14], (emphasis added).

In short, like a fish remarking to a friend, ‘what’s water?’, we are so embedded in our numerate society that much of the conceptual richness of our specific numeral system is invisible to us, and our use of mathematical notation in our research nomenclature can exacerbate this issue. While sensitive historical work can often ameliorate these interpretative issues, these habits extend into and shape education and psychological research fostered by lay familiarity with such numerals and the reliance on such notation in research. Much psychological research concerns the ‘increased cognitive simplicity offered by decimal notation’ [15, p. 290] and ‘the decimal advantage… in the accuracy of magnitude representations’ [16, p. 874]. Exponential and power notation is often invoked in describing how ‘the decimals’ are structured, leading to claims that ‘decimal fractions may thus be considered *just an extension of natural numbers*’ [17, p. 1] (emphasis added). The studies themselves predominantly address the accuracy of magnitude representation and rational number denotation through number-line estimation tasks [18–22], while studies concerning magnitude comparison between numerals [23–26] explain performance by virtue of a notation’s facility to more precisely access magnitude representations. While these are important functions that non-integer notational systems achieve in the industrialized world, they privilege precision and accuracy as their primary functions and bear little sensitivity to the system’s historical development and purpose—developments and purpose that could better inform psychological research.

It is in this context that we offer this paper as a tonic. Not as a contradiction of a well-formed orthodox position, but rather as a means to shed light on a conceptual blind spot engendered by our familiarity with integer and non-integer numerals and the widespread use of exponential notation in our research nomenclature—particularly in interdisciplinary research, in which results and terminology are imported across fields, sometimes losing their nuance, and tacitly affect the research agenda. This blind spot manifests in the subtle, often unacknowledged assumption that the development of positional notation for non-integers—‘the decimals’—was a natural, even inevitable extension of place value notation for integers. The ease with which contemporary notation blends both into a single formalism encourages a sense of conceptual continuity, reinforced by the exponential definitions that dominate both educational and scientific discourse. This blind spot yields three entailments that feel intuitive given our current notation but obscure historical and conceptual complexity:

(i) that the extension of positional notation to non-integers should have been relatively easy and fast;
(ii) that this extension was driven primarily by the demand for precise and complete non-integer denotation; and
(iii) that this extension simply denoted an extant universe of real numbers, the referents were already present awaiting a denotational system.

In what follows, we challenge these entailments by turning to historical, typological and cognitive evidence that complicates this narrative, and in so doing aim to shed light on this conceptual blind spot. We argue that the extension to non-integers was not conceptually trivial, nor historically inevitable, and that our default interpretive lens—grounded in contemporary notation and its affordances—obscures more than it reveals. Rather than treating the appearance of non-integer positional denotation as a simple extrapolation, we examine the distinct conceptual pressures, representational challenges and socio-historical contexts that shaped their emergence and use. Moreover, we hope to demonstrate how the exponential structure of the positional ‘base’ system—the structure we frequently take for granted through our research practices—was neither latent in the integer system nor extended to the non-integer system, but rather emerged with this notational extension using resources developed elsewhere, for different purposes, and only through this came to be explicitly incorporated in the system.

## 3. Non-integer denotation: a protracted historical development

### (a) Scaling: precision without non-integer denotation

Before examining the many intricacies of precise non-integer denotation, it is worth noting how the numeral system for whole numbers can allow for any precision that could practically be required, in contrast to claims that non-integer decimal notation improves upon the integers because it ‘can represent [continuous quantity] to any degree of accuracy’ [27, p. 288] unlike the integers which ‘are not accurate enough’ [27, p. 288]. The whole numeral system deals with pluralities of a fundamental unit. Briefly, if there is a situation in which that unit is too coarse and would require non-integer parts, one can just pick a smaller unit suitable for the level of precision required. We see this frequently in metrological situations: miles, yards, feet, inches, barleycorns (for discussion of the development and formalization of metrological systems in the context of linear extent, see [28]). One can move to whatever measure meets the required granularity (or mix such measures, or change the scale in a systematic measurement system; see [29] for discussion of ‘base’ and mixed systems beyond concerns of granularity), since in any specific, practical situation, there will be a minimal such unit. Our practical ends, whether in measurement, building or tracking, always have a finite granularity: even the construction of the particle accelerator at *CERN* must have used some finite approximation of π, and yet the particles still collide. The limitations of such pragmatic changes of scale or unit do not owe to a practical imprecision, but to their ad hoc character. In applications or situations in which there is no guaranteed minimal unit, in which there may be unlimited iterative partitions and divisions, or in which one needs to partition or divide some as yet unknown element, only then will such practical, ad hoc approaches relying on the whole numeral system struggle.

### (b) Ad hoc denotation: the power of fractions

While in practical settings the numeral systems for integers can get you pretty far, they evidently have limitations. To surpass such limitations and to deal with non-integers, different systems have been developed including an array of fractional systems and the decimal system with which we are generally concerned. Owing to our routine interactions with non-integer decimal notation, we often view the decimal system of denotation as the *sine qua non* of arbitrary systematic precision. In fact, adults in industrialized cultures have a preference for using fractions in discrete contexts and decimals in continuous contexts [30,31], and this is explained on the basis of semantic alignment, i.e. that the structure of fractions aligns with the structure of discrete contexts and the structure of decimals aligns with continuous contexts.

This supposed alignment is worth inspecting more closely, though. In any pragmatic setting, a setting in which we must actually operate with the (necessarily finite) denotation, the non-integer decimal system offers no great precisional advantage—any finite decimal necessarily represents a rational number, and so could be denoted in any fractional system. Computationally, in as far as we can pragmatically precisely denote with decimals, we can do so with fractional denotations. If there is no distinction in finite settings, then what is the source of this semantic alignment and is it well-founded? One key difference comes from the fact that in the non-integer decimal system, there is no minimal fundamental unit, no maximal subdivision and no longest decimal. The decimals can denote with indefinitely increasing precision (even if any specific instance or class of instances is limited). However, this is also true for the fractions. The semantic alignment, then, cannot owe to some computational account of even the potential extent or indefinite granularity of denotational precision.

It is readily apparent that the systematic manner by which the decimals achieve their denotational precision separates them from the ad hoc fractional approaches. This systematicity might appear to subsume or extend the fractional systems’ capacities. However, different fractional systems offer their own algorithmic or denotational advantages, for instance, reduced fractions (a/b) allow for easy multiplication, and Egyptian fractions (finite sums of unit fractions like 1/k+1/m) yield efficient partitions of goods.

The fractional systems’ capacities for arbitrary denotational precision may not be monolithic or consistent across contexts, but this ad hoc character should not be taken as evidence that they are somehow impoverished in this specific respect nor that this supposed lacuna requires remediation. The need for systematicity in denotation or the preferences for some practico-mathematical affordances over others are not deficits in fractional denotational systems. The longevity of fractional representations, even within systems with a positional base for integers, should give pause for thought on these often tacit views of the superiority of denoting via decimals.

### (c) Consistent denotation: fractions of powers

The computational and mathematical descriptions of fractions, decimals and their pragmatic and potential capacities notwithstanding, it is a historical fact that systematic denotation relying on fractions of powers of a number did develop. Here, we briefly examine some cases of extension of a positional notational system for integers to fractions of powers for non-integer parts, so that we might understand the contexts and limits of such extensions—extensions that the conceptual blind spot renders unremarkable.

Proust [32], through synthesizing analysis of many tablets, demonstrates how the sexagesimal place value notation (SPVN) is only invoked for non-integer parts in calculation contexts—specifically contexts requiring multiplication and reciprocals, not addition or subtraction—and these outputs are then rendered back in the standard additive metrological notations that denote non-integer parts with a fractional notation. One supposed denotational limitation of the SPVN in non-integer contexts is the lack of a sexagesimal separatrix, thus rendering magnitude marking impossible and, accordingly, accurate addition. However, Proust invites us to re-imagine this not as a limitation but as an orientation to a different purpose: ‘numbers in place value notation are not quantities, they only serve as tools for multiplicative calculations and calculations of reciprocals’ [32, p. 272]. While for us it is difficult to separate the notational system from the purpose of precisely denoting quantities, this does not appear to be an essential feature of such systems, even for those that appear to denote non-integers by fractions of powers of a base. Similarly, Chemla [33] demonstrates that even the use of positional systems in iterative calculation contexts is not monolithic and its specific, contingent applications yield results that denote differently: either factor by factor or digit by digit (positional numerals), and thus are suitable for different purposes.

While the above case references extensions of positional systems to handle non-integer parts in a manner foreign to our industrialized mode, we can look to the Arabic world of the Middle Ages for a development of a decimal system isomorphic to our own. The earliest known treatise on the Indo-Arabic^3^ ciphered positional numeral system for the whole numbers in the Arabic world is generally credited to al-Khwarizmi *ca* 820 CE [3]. At this time, finger reckoning, rhetorically expressing unknowns and (what we would now call) powers in words, alphabetic ciphers for writing numbers were dominant, as well as an alphabetic, sexagesimally anchored^4^ system (falling short of base^5^) for fractions used largely by astronomers [34, p.8]. al-Khwarizmi’s contribution was to introduce the nine digits and the cipher for zero, and their algorithms (designed for dust-board calculations that required repeated erasure and replacement of notation) for various arithmetic procedures. It is not until al-Uqlidisi in 952 CE that we see the first recognition of the use of using decimal fractions [34]. However, his usage is limited to cases of iteratively increasing an amount by one-tenth by repeatedly

[rewriting the numeral] below, brought one place down, marking the units place. It becomes13511135. We add them and get 1485 [34, p. 114]

and to cases of halving:

in what is drawn on the principle of numbers, the half of one in any place is 5 before it… the units place becomes tens to what is before it… the units place becomes hundreds in the second time of halving. So it goes always [34, p. 110].

al-Uqlidisi, then, operates with decimal fractions rarely (but adeptly), and understands how the positions relate to each other in the non-integer context just as they do for the integers. Yet they are only presented in limited calculation contexts, and generally, other non-integer denotations and manipulations are used. It appears that the power of using powers of the base of the whole numeral system, either for consistent denotation or iterative calculation, is not immediately grasped even after some situated use is present.

Only after al-Samaw’al (*ca* 1150 CE, fully 300 years after al-Khwarizmi’s introduction of the Indo-Arabic system of numerals) do we see the application of decimal fractions as a consistent and powerful denotational system: ‘until then, one only encounters, at best, an intuition still submerged in an empirical practice’ [35, p. 116]. al-Samaw’al was an excellent mathematician and a member of the *al-Karaji* school of Arabic algebraists. It was only in the context of iteratively extracting increasingly accurate approximate roots of integers that al-Samaw’al came to develop and apply a system of decimal fractions, a system which he then re-applies to arithmetic outside of this context [35].

### (d) Denoting with decimals

**Figure 2.**
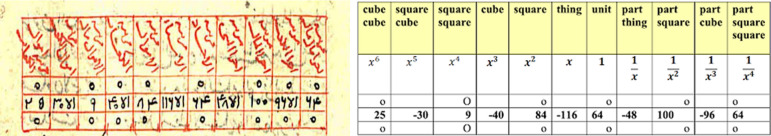
Left: al-Samaw’al’s extraction of the square root of polynomials as produced in al-Bāhir (left). In the absence of such tabular symbolism such polynomials were expressed verbally, with the grouping of additive and subtractive terms, and required rhetorical, geometric and other methods for their representation and manipulation. The tabular symbolism developed by al-Samaw’al allowed him to further the application of arithmetic to expressions with unknowns, specifically in the cases of subtractive quantities and unknown parts: what we now term negative coefficients and negative exponents. Right: al-Samaw’al’s table of extracting root square translated (reproduced from [36]). The second row of this translated table is not present in al-Samaw’al but is presented for clarity for the modern reader (see [36, pp. 78−83] for details of the table construction and translation, as well as the root extraction procedure).

Although al-Samaw’al recognized the power and use of using decimal fractions, his application was still limited to algebraic numbers: numbers that emerge from algebraic operations like multiplication, division, root extraction, etc. It is with al-Kashi *ca* 1429 CE that we see the maturation of the decimal system used to approximately represent transcendental numbers like π. Moreover, the manner of denotation and manipulation that al-Samaw’al deploys to work with these decimal fractions is not a simple extension of the Indo-Arabic positional numeral system. Rather, he ‘[adopted] a notation previously used for the case of polynomials in general to obtain the decimal representation of any algebraic number; and lastly, apply the operations elaborated earlier for polynomials^6^ in the widest sense to these representations so as to obtain at the same time the rules for calculating fractions’ [35, p.119]; a notation, it bears mentioning, tabular in construction, relating integers with rhetorical powers of an unknown, whose skilled manipulation enacted what we would now describe with the law of exponents (figure 2). Again, it is not until *ca* 300 years after al-Samaw’al, and more than 500 years after the introduction of the Indo-Arabic decimal system for integers, with al-Kashi, that we see a fully mature system of decimal fractions, with a common denotation for integers and parts, and a convenient separatrix, alongside a clear and thorough understanding and discussion of the use, operability and foundation of the decimal system [37].

Looking to Renaissance Europe for another decimal development, we see a similarly protracted trajectory. In the high Middle Ages, the Indo-Arabic numeral system first reached Europe, with Leonardo of Pisa’s *Liber Abaci* (1202 CE) being the first explication of the system and its methods, again occurring in a background of finger reckoning, fractional parts and commercial fractional systems, as well as a sexagesimally anchored system used in astronomical and trigonometric settings. One must wait until 1585 with Simon Stevin to first see a treatise describing the decimal fractions, with a consistent notation, in a manner akin to al-Kashi. Moreover, the widespread adoption of the non-integer decimal system of denotation—and the specific positional notation with a separatrix—does not emerge until the proliferation of the tables of logarithms owing to Napier in the early seventeenth century. In the intervening years we see ad hoc use of decimal fractions [3], with practices like ‘decimal magnification of numbers for root extraction’ [38, p. 174] similar to the metrological change of unit described above, ‘decimal magnification ($10^{n}$ or $6\cdot 10^{n}$) of the radius for the calculation of trigonometrical ratios’ [38, p. 174], or the resolution of seeming decimal/sexagesimal fractions intoa whole number of its smallest unit expressed in decimal form. ‘[Some medieval European astronomers] would then perform the calculation with the decimal integers, and only afterward convert back to sexagesimal fractions’ [39, p. 2], and other similar empirical practices approaching the decimal.

### (e) Summary

**Figure 3.**
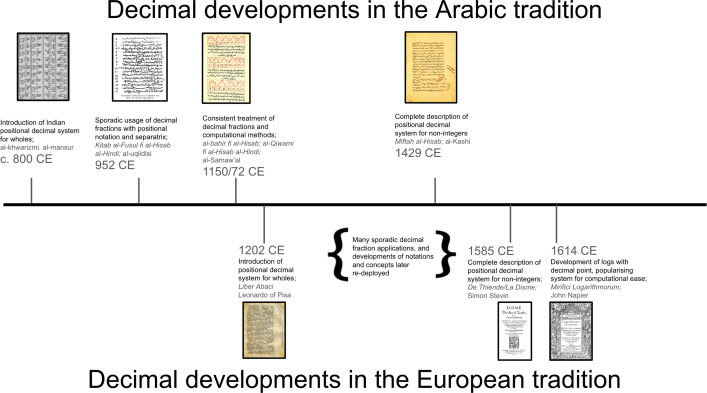
Timelines of the important landmarks in the development of the decimal base system for non-integers in the Arabic and European traditions. While the history and development of the decimal system for non-integers cannot be completely rendered in one dimension, these timelines display the long, elaborate and protracted history in the development and adoption of the decimal system for non-integers, occurring over hundreds of years, and as a result of many small steps rather than a simple extension of the decimal system for integers.

Decimal fractions and a coherent positional notation for them are relatively recent in the development of our numeral system. Their development and adoption took hundreds of years from the adoption of positional numeral notation for whole numbers (figure 3). It appears that other systems—which were adequate for the purposes to which they were put—did not present a need, or were not readily adaptable, to make such a leap. This contrasts with the first entailment of this conceptual blind spot:

(i) that the extension of the positional system to non-integers should be *was not* relatively straightforward and fast.

Moreover, the incorporation of exponents into the structure of these decimal denotations was borrowed from algebra, the arithmetic of unknowns and even then these exponents were still in a rhetorical mode or using tabulation, as opposed to the integer indexes with which we are now familiar. It is only during the time periods of the extension of this positional system, with tools from other mathematical domains, that we begin to see exponentiation applied to concrete numerals, let alone to the structure of numeral systems generally. This highlights that the continuity of the non-integer and integer notational systems was constructed through these extensions rather than as an explication of the integer system’s latent structure, in contrast with our usage of exponents in typologizing these systems and defining ‘base’.

## 4. Denoting non-integers: a diversity of developments and domains

### (a) Denotations emerging inconsistently

While there is a certain consistency to the fact that the non-integer decimal system took a long time to develop after the introduction of the Indo-Arabic decimal numeral system for integers, there is not a concomitant consistency in the contexts within which the systems were developed or adopted. As mentioned previously, al-Uqlidisi’s treatment of decimal fractions was limited in scope and appeared in specific contexts of calculation: halving and increasing by a tenth. It was divorced from concerns of arbitrarily precise denotation, and was expressed via analogy with the local values of the whole numeral system and with patterns in computations in such notation, more akin to a contextually useful change in scale or granularity rather than a systematic innovation. Separately, but still in the Arabic tradition, al-Samaw’al’s more mature development of decimal fractions occurred in the context of extracting roots by iterative algorithms; his introduction of the decimal system occurred in the chapter immediately following a chapter concerning root extraction [37, p. 377]. It was only after this with al-Kashi that the system of denotation and calculation was extended to approximating other arithmetic values and, later, irrationals generally. Rather than beginning from requiring a system in which everything could be arbitrarily precisely denoted through iterative procedures, the development of a non-integer decimal system in the Arabic tradition emerged almost in reverse, whereby specific iterative algorithms yielded a denotational system that could represent their results, and this was then extended to other denotational or arithmetic situations.

Separately, in the European tradition, we see again a different context of decimal development. In Stevin’s introduction of the non-integer decimal system, he is explicit about the algorithmic and commercial purpose of the system, opening the pamphlet with *‘La Disme: Teaching how all computations that are met in business may be performed by integers alone without the aid of fractions’* [38, p. 219]. Addressing the pamphlet to a host of practical, numerate professions, he offers his system, stating that ‘If by these means, time may be saved which would otherwise be lost, if work may be avoided, as well as disputes, mistakes, lawsuits and other mischances commonly joined thereto, I willingly submit La Disme to your consideration’ [40 p.22]. After briefly introducing the decimal algorithms for addition, subtraction, multiplication and division, and justifying their accuracy, Stevin then indicates the use of the system in practical situations that each profession might encounter. In this pamphlet, Stevin is solely occupied with both practical ends and practical computations. He offers his system as a means to avoid labour and mistakes. In the practical appendix, when dealing with surveying, he is even sensitive to the fact that the results of his decimal representation must be cashed out in the measures of the day (e.g. feet, inches) ‘but this is a thing which the surveyor must do but once, i.e. at the end of the account which he gives to the proprietaries’ and that the level of precision of the decimal result might be unnecessary ‘as the majority think it useless to measure the smaller units’ [40, p.30]. The primary motivation for Stevin is ease of computation, not of precise denotation nor of algebra or dealing with unknowns. While his development of this system, and the context in which he developed it, is not exclusively captured by this didactic pamphlet, it does speak to the perceived import and purpose of such a system.

### (b) By dint of a point? Numerous notational options

As one might expect from such a diversity of developmental contexts and traditions, the notations for the decimal system were also myriad. Returning to al-Samaw’al and his decimal fractions, originally ‘elaborated within the context of the problem of the extraction of the *n*th root of a number and problems of approximation’ [35, p. 124], we can look to the tools and methods of this elaboration. In particular, al-Samaw’al relied on methods for algebra, the arithmetic of the unknown. al-Samaw’al, moving beyond solely rhetorical methods, had introduced a tabular symbolism and method for performing arithmetic operations on such combinations of unknowns. It was on the basis of this algebraic tabular system that al-Samaw’al initially operated with and denoted decimal fractions: ‘As we can see this notation is based on the following principle: to separate the integer part and represent the fractional part according to al-Samaw’al’s technique also used in his Algebra for representing a polynomial’ [35, p. 122].^7^ However, this tabular representation was not readily pronounceable and so ‘[t]o get round this difficulty, al-Samaw’al was inspired by a notation then used for ordinary fractions and relates the fractional part to the same denominator’ [35, p. 123]. Thus, there is a seeming divorce between the notation for calculation and that for denotation or pronunciation, not all too dissimilar from the divorce between Stevin’s system for calculation and its translation into the measures of the practitioners or from the Babylonians’ operational and practical representational systems. Again, it is not until al-Kashi, *ca* 300 years later, that we see the computation and denotation sharing the same symbolism, extending the positional system of the integers to the decimal fractions with a convenient separatrix.

In the European tradition, we also see some diversity of notations for systems of decimal fractions before the convenient notation with which we are now familiar emerged. Stevin’s first attempts appear to us now to be unnecessarily cumbersome, especially given his overall grasp of the power of the system. He writes 32 and 57/100 as 32(0)5(1)7(2). He appears to miss that the positional system requires only some marker of magnitude like a decimal separatrix or a single numeral representing the minimum partition of ten (or exponent in our modern scientific notation). Instead, he uses this circular notation, a notation he also uses in his algebraic work in which 2(2)+3(1) represents what we would now write as $2x^{2}+3x$; a notation he owes to Bombelli (d. 1572) [41], but also developed in the same manner by Chuquet (d. 1487) [3]. The development of notation for powers originated in algebra, in a move away from the rhetorical mode and with the adoption of numerals (as opposed to distinct glyphs or words) for expressing powers of an unknown, and was only later applied to concrete numerals. This notation, and perhaps also an exaptation of this exponential denotational structure, was then borrowed by Stevin in the context of the decimal system for fractions. Moreover, during this period in Europe there were countless competing notations for systems of decimal fractions including the use of Roman numerals in a manner similar to Stevin: $32^{0}5^{i}7^{ii}$; typographic modifications of the fractional part of the numeral: $32^{\underline{57}}$; the use of decimal separatrix isomorphic to our own: 32|57; using a numeral to express the order of the final atomic numeral: 3257(2) and many others [38,42]. These competing notations extend the positional system for integers to the non-integers to greater and lesser extents, and rely on different foundations of powers, positions and fractional expressions. The notation for the decimal system of fractions was not developed solely as an extension of that for the integers, it owed its notation to innovations elsewhere, and even after its computational structure was solidified still had representations that were structurally distinct.

### (c) Notation bene: adoption of decimals

So far, we have seen that there are manifold different extensions of the ciphered-positional systems to handle non-integer parts, and that they often emerge in different contexts with different tools, and in competition with other fractional systems. Further, those who advocated for the non-integer decimal system with which we are now familiar often pointed to an apparent computational ease, either through its capacity for indefinite iteration and approximation or owing to its rendering of all computations into the simple integer calculus. At no point, do we see a simple appeal to precision or concerns over the lack of such in the competing systems. Computation is king; but we must ask, what computations?

For instance: 0.375×0.16 is a relatively difficult multiplication problem, requiring multiple steps, carrying digits and managing the placement of the decimal point. However, using the corresponding fractional denotation 3/8×4/25 is pretty easy, it is 12/200, or 3/50=0.06. The decimal system is not some panacea. While it does render all arithmetic problems in the same language, this comes at the cost of some algorithmic complexity. However, 3/8+4/25 is altogether more complicated than 0.375+0.16, which is readily apparent as 0.535, or 107/200 when expressed as a reduced fraction. This is not only born out computationally, but also in actual human performance, with people’s representational choices often affected by operational rather than semantic alignment [43]. While many used decimal fractions in an ad hoc fashion, and Stevin and al-Kashi gave clear treatises concerning their use in commerce and mathematics, ‘[w]e have to await the elaboration of the logarithmic function, notably with Napier, for decimal fractions to be integrated into a mathematical corpus effectively practised’ [35, p. 132]. The logarithmic tables that Napier produced, and Briggs later improved, were specifically designed in order to convert onerous multiplication and division problems into problems of addition and subtraction within the positional-ciphered system. Anachronistically, log⁡(A×B)=log⁡(A)+log⁡(B), so: to find A×B, one found A and B in the index of the logarithmic tables, computed the sum of their logarithms—an easy task in a decimal system—found this sum in the logarithm tables, and the index of this sum in the table would be the value A×B. Such was the use and ease of this system that Laplace, an eminent eighteenth century mathematician and engineer, said that the logarithms, ‘by shortening the labours, doubled the life of the astronomer’ [44, p. 109]. The popular adoption of the decimals was not owing to concerns over precise denotation nor to a comprehensive computational superiority, but to the existence of a specific context and arithmetic method in which the computational advantages of the system were immensely beneficial.

### (d) Summary

Not only did decimal fractions and a coherent notation for such develop over a protracted historical period, but the innovations were drawn from diverse domains and for distinct purposes. The purposes for the developments owed to interests in the distinct domains of algebra and iterative algorithms, commerce and computational ease, as well as logarithmic tables and astronomical calculation. The notations and concepts for the developments were not solely driven or primarily accepted due to improvements in denotational precision, but were adapted from algebraic domains. This contrasts with the second entailment of the conceptual blind spot:

(ii) that the extension of the positional numeral system to non-integers was motivated by a concern with precisely deneting extant quantities *by particular computational practices and adopted owing to contingent utilities*.

Moreover, these notations for expressing non-integers were borrowed from innovations in expressing exponents—innovations that occurred in the algebraic domain to deal with the repeated multiplications of unknowns. These notations were only then carried into the structure of the numeral system through the extension of the positional system to the non-integers, rather than endogenous explication of the integer system’s structure. It is through this extension and borrowing of notation that we come to describe the numeral system as structured by exponentials, exponentials which could be positive, negative or zero. Although we now use exponents to define and communicate about ‘base’ as a concept and systems with a ‘base’, this framing was neither obvious nor inevitable, and emerged as developments in diverse domains were combined and cross-pollinated. To understand the history of the ‘base’ concept proper and the systems it structures, we must remain cognisant of the manner by which we developed these tools.

## 5. Metaphysical shifts

### (a) Notation shaping ontology

The extension of the positional system to the non-integers generally, and the tools for its construction, engendered new questions and capacities. Humans cannot interact with numbers directly, being non-spatiotemporal objects, and instead come to learn and describe their properties by interacting with numerals, concrete representations of numbers. We cannot take for granted that the system of representation is some transparent medium, but rather that its composition affects how we conceptualize numbers beyond just affording certain interactions or reducing the computational load of others. It might now seem apparent to us that 1/2=0.5 and $\sqrt{2}=1.414\ldots$ are both equally valid numbers, or even $\sqrt{2}$ and π=3.14…. However, the question of a shared ontology for rationals, irrationals and transcendentals was a long and hotly debated topic within mathematics. The length of time it took to extend the decimal fractional system even from algebraic numbers with al-Samaw’al to transcendental numbers like π by al-Kashi serves as an indication of the breadth of the gap that was bridged, a gap which was not bridged wholesale as might be expected if there were extant referents simply awaiting denotation. Perhaps then it should not come as a surprise that it was also Stevin who, in a separate treatise *l’Arithmetique*, gave the first lucid explanation of the shared ontology of the integers and continuous magnitudes [38]. While not a necessary cause, the shared denotational form for the non-integers both demonstrated the arbitrary divisibility of whole numbers, represented integers and magnitudes in the same manner and provided tools that allowed for equivalent algorithms for both discrete and continuous magnitudes in this representation. In some sense, if it looks like a duck, and quacks like a duck, it is hardly surprising that some then come to think of it as a duck.

The system of representation also introduces new questions, for instance, concerning numbers with an infinite decimal representation. While many systems can allow for unending representation, e.g. 21/21/41/9…, the decimal fractional system is the only one that has this iterative, potentially infinite, representation baked in. Even from Stevin’s first description of the system, though he only ever used finite decimals, he was aware at least of the plausibility of infinite repeating decimals, even if not relevant to his ends (see Proposition 4 of Stevin in [40, p.27-28]). However, how one should work with these infinite representations of finite quantities (and if we should truly think of them as representing anything) proved to be a challenge to the mathematical community for centuries to come, eventually relying on advanced machinery of limits, Dedekind cuts, ultrafilters, etc. Even now, there is an entire Wikipedia page, and countless blogs, textbook chapters and mathematics education papers dedicated to the question of whether and how 0.999…=1 [45].

### (b) Summary

The introduction of decimal fractions and a coherent positional notation for them did more than simplify certain computations and algorithms or improve numerical denotation. These new representations introduced new metaphysical and mathematical problems, which, in turn, serve as motivation for further mathematical developments. We briefly mention these ontological changes and challenges as a reminder that our conception of what numbers are, and how numerals denote, is the result of a long intellectual tradition. For instance, even the idea of extending the positional system to represent the non-integers takes for granted that there are numbers which could even be represented, let alone should be represented by the same system—a stance that only emerges after this innovative extension was completed. This contrasts with the third entailment resulting from our conceptual blind spot:

(iii) that these new referents were extant and requiring a denotational system *came to be treated as numbers only as a result of these innovations*.

## 6. What is the point?

As mentioned throughout this article, and this special issue generally, the concept of ‘base’ is more complex than it may seem. Although as researchers we frequently invoke exponentials in the discussion of the concept of ‘base’ and are liable to treat ‘base’ in integer and non-integer systems equivalently:


(6.1)am⋯a0:=∑k=0m(ak×10k),(6.2)am⋯a0.a−1⋯a−n:=∑k=−nm(ak×10k).

The historical trajectory of the extension of positional notation to the non-integers and the delayed invocation of exponentiation into their structure indicate that this may be inappropriate. This non-integer extension relied on methods and concepts external to number denotation, rather than elaborating endogenous structure. This non-integer extension was not wholesale but gradually became more expansive over time, rather than emerging from the identification of some latent structure and its broad application. This non-integer extension was adopted owing to contingent computational concerns, rather than the elaboration of a representational principle. This non-integer extension altered our concept of the actual referents of these notations and how we perceive objects like algebraic, irrational and transcendental numbers. Rather than extending the exponential structure of equation (6.1) to the non-integers in equation (6.2), the exponential structure emerged with the development of equation (6.2), owing to changes in diverse mathematical domains, and only now can we look back and describe ‘base’ and numeral systems using the exponential structure of equation (6.1). In short, while modern users of the Indo-Arabic positional system might describe and conceptualize its ‘base’ in terms of exponentials, the history of its extension to the non-integers indicates that this is contingent, contemporary and hard-won. To understand the development of the non-integer system, integer systems, the concept of ‘base’ itself and even how modern people do and should develop such skills, we need to remain aware of this protracted, contingent history.

There’s an old joke about a tourist visiting Ireland. They watch a labourer cutting paving stones by hand with an angle-grinder. ‘In my country,’ the tourist calls out, ‘we have a laser-guided machine that makes those cuts with a precision of one-thousandth of a millimetre.’ The labourer replies, deadpan: ‘That’d never fly here. No, here we have to get it *just right*.’ Our present-day familiarity with numerals, their structures and their purposes can leave us in the position of the tourist, judging a system of practice based upon our contemporary machinery and goals. Precision and exponential structure may now define how we use and talk about ‘base’ systems and non-integers, but they did not define how such systems developed. If we let these modern frameworks obscure their contingent, computational origins, we may fail to get our theories of number, ‘base’ and notation *just right*.

## Data Availability

This article has no additional data.

## Footnotes

^1^
We will use the term *exponential*, *exponent* and *power* synonymously throughout this text. This is consistent with mathematical practice [5] as in the phrase ‘2 to the power of 3 is 8’ where 3 is the *exponent* or *power*.

^2^
We will follow this usage throughout, where the plural *decimals* will always refer to the decimal system for integers extended to the non-integers, in keeping with the vernacular.

^3^
Although many of the authors and commentators have used different terms, and the term Indo-Arabic can be, at times, anachronistic, we use it throughout to refer to the structure of a ciphered positional numeral system for clarity of reference at the cost of some historical accuracy.

^4^
The system for degrees (the integers in this notational system) was alphabetic, not positional, called *abjad* or *jummal* notation. Only in the partitioning of the degree into minutes, seconds, etc. do we see the use of a sexagesimal anchor and positionality in the notation (though still *jummal*, generally ranging from 1 to 59). Thus, rather than being a true notational base or ciphered-positional system, it was rather a specific technique for dealing with non-integer parts. For further discussion, see [8, ch. 7].

^5^
See Pelland [2] in this issue for the conceptual distinction between a compositional anchor and a base.

^6^
Rashed [35] uses the term polynomial, but it is important for the modern reader to remember that this algebra was still being written in the rhetorical mode, with distinct words for each power of an unknown. e.g. square, cube, square-square, square-cube.

^7^
The ‘Algebra’ mentioned in this quote is al-Bahir fi’l-Jabr, ‘*The brilliant in algebra*', written by al-Samaw’al in approximately 1150 CE.
